# β-1,6-Glucan From *Pleurotus eryngii* Modulates the Immunity and Gut Microbiota

**DOI:** 10.3389/fimmu.2022.859923

**Published:** 2022-05-02

**Authors:** Xue Wang, Yunhe Qu, Yuan Wang, Xiang Wang, Jialei Xu, Hailing Zhao, Donglin Zheng, Lin Sun, Guihua Tai, Yifa Zhou, Hairong Cheng

**Affiliations:** Engineering Research Center of Glycoconjugates, Ministry of Education, Jilin Provincial Key Laboratory of Chemistry and Biology of Changbai Mountain Natural Drugs, School of Life Sciences, Northeast Normal University, Changchun, China

**Keywords:** β-1,6-glucan, *Pleurotus eryngii*, immunoregulation, gut microbiota, SCFAs

## Abstract

Polysaccharides from *Pleurotus eryngii* exhibit a variety of biological activities. Here, we obtained a homogeneous branched β-1,6-glucan (APEP-A-b) from the fruiting bodies of *P. eryngii* and investigated its effect on immunity and gut microbiota. Our results showed that APEP-A-b significantly increases splenic lymphocyte proliferation, NK cell activity and phagocytic capacity of peritoneal cavity phagocytes. Furthermore, we found that the proportion of CD4^+^ and CD8^+^ T cells in lamina propria are significantly increased upon APEP-A-b treatment. Additionally, APEP-A-b supplementation demonstrated pronounced changes in microbiota reflected in promotion of relative abundances of species in the Lachnospiraceae and Rikenellaceae families. Consistently, APEP-A-b significantly increased the concentration of acetic and butyric acid in cecum contents. Overall, our results suggest that β-1,6-glucan from P. eryngii might enhance immunity by modulating microbiota. These results are important for the processing and product development of P. eryngii derived polysaccharides.

## Introduction

*Pleurotus eryngii* (*P. eryngii*, known as king trumpet mushroom or king oyster mushroom) has been extensively used in Europe, the Middle East, North America and Asia as an edible mushroom for a broad spectrum of nutritional benefits and pharmacological effects as anti-inflammatory, antioxidant, antitumor and antibacterial agents, as well as hepatoprotective and hypolipidemic activities (1, 2). These effects are likely due to the many active ingredients in *P. eryngii*, such as bioactive polysaccharides, peptides, and sterols (3). In particular, polysaccharides are the dominated contributors that are reported to exert antitumor (4), anti-inflammatory (5) and immunological activities (6). In the past 20 years, the structural characteristics of polysaccharides derived from fruiting bodies and mycelium of *P. eryngii* have been widely explored (2, 6, 7). The linear (1→6)-β-glucan (8), branched (1→3)-β-glucan (9), 3-O-methylated heterogalactan (6) and heteropolysaccharides (10) have been extracted and purified from *P. eryngii* fruiting bodies. However, few studies on branched β-1,6-glucans from *P. eryngii* have been reported.

β-1,6-glucan is an important component of fugal cell walls (11, 12) and accounts for 5% to 21% of its mass depending on the species (13). This glucan has been isolated and purified from fungi, e.g. *Pleurotus florida, Lentinula edodes, Agaricus brasiliensis, Agaricus brasiliensis*, and *Amillariella mellea* (14–16). β-1,6-glucan has a variety of biological activities, including immunomodulatory function (14, 15), as well as anti-tumor (16) and anti-oxidant activities (17). Praloy K. Maji et al. have reported that a branched β-1,6-glucan can activate macrophages, splenocytes and thymocytes (14). β-1,6-glucans from *Agaricus bisporus* and *Agaricus brasiliensis* have a significant effect on THP-1-derived macrophages (15). Daisuke Yamanaka et al. reported that *Agaricus brasiliensis* polysaccharides with β-1,6-glucan as the main component, can induce production of cytokines from both murine splenocytes and bone marrow-derived dendritic cells (18). Furthermore, β-1,6-glucans exert their anti-cancer effects by immunomodulation rather than by direct cytotoxic activity. We previously found that the (1→6)-β-D-glucan (AAMP-A70) isolated from *Amillariella mellea* can inhibit the viability of colon cancer cells by resetting tumor-promoting M2-like macrophages to a tumor-inhibiting M1-like phenotype (16).

Several data suggest that gut microbiota is engaged in dynamic interactions with the intestinal innate and adaptive immune system, thereby affecting different aspects of its development and function (19, 20). Previous reports showed that polysaccharides can alter gut microbiota composition, which essentially contributes to attenuating colitis (21), controlling cardiovascular disease (22), and blocking cognitive impairment (23). The impact of polysaccharides on gut microbiota are commonly assessed by using culture-dependent techniques and 16S rRNA sequencing. Cultured microbes include *Bifidobacterium* spp., *Lactobacillus *spp., Enterobacteriaceae, *Enterococcus* spp., and *Clostridium perfringens*, which represent probiotics (24), opportunistic pathogens (25, 26), and pathogens (27), respectively. Based on 16S rRNA sequencing, β-1,6-glucan derived from highland barley (HBBG) can enhance the relative abundance of Bacteroidetes and Verrucomicrobia in UC mice (28). And orally taken (1→6)-β-D-glucan (GLP) from *Ganoderma lucidum* can significantly abrogate dextran sodium sulfate (DSS)-induced colitis in rats by increasing SCFA-producing bacteria, such as *Ruminococcus_1* and reducing pathogens such as *Escherichia-Shigella* (21).

Previous work in this area has mostly focused on bioactive agents from crude *P. eryngii* polysaccharides *in vivo* or the activation of immune cells by a homogeneous polysaccharide on the cellular level. Homogenously branched β-1,6-glucan fractions from *P.* eryn*gii* and their effects on immunity and gut microbiota *in vivo* have rarely been investigated. In this study, we obtained a homogenously branched β-1,6-glucan named APEP-A-b, from the fruiting bodies of *P. eryngii* and investigated its effect on immunity and gut microbiota. In previous studies, doses of 50 to 800 mg/kg·d of these polysaccharides were used to stimulate the immune system in mice (29, 30). Based on this observation, we evaluated the immunomodulatory activity from APEP-A-b doses of 100, 200 and 500 mg/kg·d. Our results will contribute to understanding structure-activity relationships of these polysaccharides and provide a foundation for development of β-1,6-glucan as a therapeutic agent to enhance immunity.

## Materials and Methods

### Materials and General Methods

Fruiting bodies of *Pleurotus eryngii* were purchased at the local market in Jilin Province, PR China and were identified by using rDNA-ITS sequencing analysis. ConA, LPS, MTT, pepsin, α-amylase, gastric lipase, trypsin, gastric lipase, bile salt, and SCFA standards, including acetic, propionic and butyric acid were purchased from Sigma (Sigma Aldrich, St Louis, MO, USA). DMEM High Glucose Medium and RPMI-1640 were purchased from Gibco (Gibco, Grand Island, NY, USA). Penicillin/streptomycin was obtained from the Tina JinHao Yang Biological Manufacture Co., Ltd. (Tianjin, China). Fluorescent microspheres were purchased from Thermo Fisher Scientific (Hudson, NH, USA). All other reagents were of analytical grade or better.

### Preparation of Polysaccharides from *P. eryngii*

Fruiting bodies were initially extracted with distilled water at 100 °C for 4 h. This procedure was repeated twice, and the resulting products were dried and extracted using 0.5 M NaOH (1:20, w/v) with trace amounts of NaBH_4_ at 80 °C for 3 h × 3. Extracts were neutralized with glacial acetic acid, dialyzed against tap water for 48 h, concentrated under vacuum, and freeze-dried to obtain the total polysaccharides. These were applied to a DEAE-cellulose column (8.0 × 20 cm, Cl^-^), eluted first with distilled water and then with 0.4 M NaCl. The fraction eluted by 0.4 M NaCl was further purified using gel-permeation chromatography (Sepharose CL-6B) to give the final fraction APEP-A-b. Total sugar content was measured using the phenol-sulfuric acid method with glucose as the standard (31).

### Molecular Weight Determination

Molecular weight distribution was measured using gel-permeation chromatography with a TSK-gel G-3000PWXL (7.8 × 300 mm; Tosoh Corporation) connected to a Shimadzu HPLC system as described by Zhang et al. (32). The molecular weight of the polysaccharide was determined using a standard curve pre-calibrated with standard dextrans (50, 25, 12, 5 and 1 kDa).

### Monosaccharide Composition

Monosaccharide composition was assessed by using high performance liquid chromatography (HPLC) (32). Briefly, polysaccharide samples (1 mg each) were hydrolyzed at 80°C for 16 h in 1 mL anhydrous methanol containing 2 M HCl and then by using 1 mL 2 M trifluoroacetic acid at 120 °C for 1 h. After derivatization with 1-phenyl-3-methyl-5-pyrazo-lone, derivatives were analyzed by using a Shimadzu HPLC system.

### Methylation Analysis

Methylation analysis was performed using the assay of Needs and Selvendran (33). Polysaccharides were dissolved in DMSO (0.5 mL) and methylated using a mixture of NaOH/DMSO (0.5 mL) and iodomethane (1.0 mL). The reaction was extracted with CH_2_Cl_2_, and then the solvent was removed by vacuum evaporation. Complete methylation was determined by disappearance of the -OH band (3,200-3,400 cm^-1^) in the FT-IR spectrum. The per-O-methylated polysaccharide was later hydrolyzed using HCOOH (85%, 1 mL) for 4 h at 100°C and then CF_3_COOH (2 M; 1 mL) for 6 h at 100°C. Following reduction and acetylation, the resulting alditol acetates were analyzed by gas chromatography-mass spectrometry (GC-MS, 7890B-5977B, Agilent Technologies, Inc.) coupled to a DB-35 ms capillary column (30 m × 0.32 mm × 0.25 mm).

### NMR Analysis

^1^H-^13^C HSQC and HMBC NMR spectra were acquired at 20°C on a Bruker Avance 600 MHz spectrometer (Bruker) with a 5 mm broadband probe operating at 600 MHz for ^1^H and 150 MHz for ^13^C. Polysaccharides (20 mg) were dissolved in D_2_O (0.5 mL) and centrifuged to remove precipitates. Chemical shifts are given in ppm (δ) relative to the resonance from acetone at δ 2.17 and 29.19 corresponding to ^1^H and ^13^C signals.

### Animal Experiments

Healthy female C57BL/6 mice (six weeks old) were purchased from Vital River Laboratory Animal Technology Co., Ltd (Beijing, China). Mice were housed under pathogen-free conditions and allowed access to food and water *ad libitum*. Mice were randomly divided into four groups (n = 6), including Control group, 100 mg/kg·d of APEP-A-b group, 200 mg/kg·d of APEP-A-b group and 500 mg/kg·d of APEP-A-b group. Distilled water or APEP-A-b were intragastrically administered for 14 days. On day 15, mice were sacrificed to assess macrophage phagocytic capacity, lymphocyte proliferation and splenic natural killer (NK) cell activity. Cecal contents were also collected for gut microbiota analysis. All animal experiments were carried out in compliance with the Animal Management Rules of the Ministry of Health of People’s Republic of China and approved by the Animal Care and Use Committee of Northeast Normal University.

### Spleen Weight and Index Determination

Mice were sacrificed by cervical dislocation, and spleens were removed and weighed. The spleen index was calculated as the ratio of immune organ weight to body weight.

### Phagocytic Capacity of Macrophages Assay

After the mice were sacrificed, peritoneal cells were harvested by peritoneal lavage with 8 mL of cold PBS. Cells were collected by centrifugation at 1000 rpm for 10 min and seeded onto 6-well plates at a density of 8 × 10^5^ cells per well, followed by incubation for 2 h. Fluorescent microspheres were prepared following the manufacturer’s instructions and adjusted with 1% of BSA to about 5 × 10^7^ microspheres per mL. Subsequently, 100 μL of beads was added to each well and held at 37°C for 90 min. Cells were washed with PBS three times and collected. The phagocytic capacity of macrophages was assessed by using flow cytometry. The rate of phagocytosis was calculated using the following equation: phagocytosis rate (%) = number of macrophages that were phagocytosed beads/total number of macrophages ×100%.

### Lymphocyte Proliferation Assay

T cell and B cell proliferation was determined using the MTT assay. Briefly, the splenocyte suspension was adjusted to a concentration of 5 × 10^6^ cells per mL using complete RPMI-1640 medium and was then seeded in 96-well plates (100 μL per well) with 100 μL Con A (final concentration was 5 μg/mL) or LPS (final concentration was 10 μg/mL) per well as mitogens for T and B lymphocyte. The plate was incubated for 72 h under 5% CO_2_ and a humidified atmosphere at 37°C temperature. Following the 72 h of incubation, 100 μL MTT (5 mg/mL) solution was added to each well, and the microplate was incubated for 4 h and then 50 μL SDS was added per well to dissolve formazan crystals. The absorbance was measured at 570 nm using a microplate reader.

### NK Cytotoxicity Assay

Splenocytes (1 × 10^6^ cells per well) and YAC-1 cells (4 × 10^4^ cells per well) were incubated in 96-well plates. Mixtures were incubated under 5% CO_2_ and a humidified atmosphere at 37°C temperature for 4 h. Culture supernatants were then collected by centrifugation at 250 g for 5 min and were mixed with LDH solution. The absorbance at 492 nm was measured using a microplate reader. The percentage of NK cell cytotoxicity was calculated according to the following equation:


Cytotoxicity (%) = (ODexperimental-ODcontrol)×100%/(ODmaximum-ODspontaneous).

### Cell Isolation and Flow Cytometry Analysis

Isolation of colonic lamina propria cells was performed using a previously established method (34). In brief, luminal content and extraintestinal fat tissue were removed, and colons were cut into 0.5 cm pieces that were initially incubated with HBSS (without Ca^2+^ and Mg^2+^) containing 5% FBS, 2 mM EDTA, and 1 mM DTT to remove epithelial cells and mucus. This was then digested in PBS solution containing 5% FBS, 1.5 mg/mL collagenase VIII, and 0. 25 mg/mL DNase I. The digested cell suspension was then washed with PBS solution and filtered with a 45-μm cell strainer. Antibodies used for colonic lamina propria staining included CD3(FITC), CD4 (PE), CD8 (PerCP), CD45.2 (PerCP), CD19 (FITC), F4/80 (PE). Cells were analyzed using an Agilent NovoCyte Quanteon (Agilent, Palo Alto, USA).

### *In Vitro* Simulated Digestion of the APEP-A-b

The *in vitro* simulated digestion was performed according to a previous report with minor modifications (35). Briefly, the stimulated salivary fluid, gastric fluid, and small intestinal fluid were prepared.

The artificial saliva solution was prepared by dissolving 119 mg Na_2_HPO_4_, 9.5 mg KH_2_PO_4_, 400 mg NaCl and 1.9 mg α-amylase (87.4 U/mg) in 50.0 mL distilled water. The gastric electrolyte solution (GES) was prepared by dissolving 155 mg of NaCl, 55 mg of KCl, 7.5 mg of CaCl_2_·2H_2_O and 30 mg of NaHCO_3_ in 50.0 mL distilled water. Then, 12.5 mg of gastric lipase, 11.8 mg of pepsin, and 0.5 mL of CH_3_COONa were added to 50 mL of GES. The small intestinal medium was prepared by dissolving 0.27 g NaCl, 32.5 mg KCl and 12.5 mg CaCl_2_ in 50 mL distilled water and adjusting pH to 7.0 using 0.1 M NaOH solution. Then, 2.6 mg trypsin, 40 mL bile salt (4%, w/w) and 20 mL pancreatin solutions (7%, w/w) were added into 20 mL of small intestinal medium.

For the simulated digestion test *in vitro*, 4.0 mL (8.0 mg/mL) of the APEP-A-b solution was mixed equally with artificial saliva and incubated in a water bath oscillator (37°C, 120 rpm), with distilled water set as the control in a similar manner. Samples were analyzed after 0 or 6 h of digestion.

Thereafter, the pH was adjusted to 2.0 with HCl (0.1 M), and 5 mL simulated gastric juice was preheated at 37°C in a water bath, followed by the addition of 5 mL the previous stage of simulated digestive juice. The mixture was then incubated at 37 °C for 6 h. Samples were analyzed after 0 or 6 h of digestion. Next, the pH was adjusted to 7.0 with NaOH (0.1 M) to simulate small intestinal fluid. Afterward, 3 mL simulated small intestinal fluid was preheated in a 37°C water bath, followed by the addition of 10 mL the previous stage simulated digestive juice. Then, the mixture was incubated at 37°C for 6 h, and the enzyme was inactivated in boiling water. During incubation, samples were taken out after 0 or 6 h of digestion for further analysis.

### *In Vitro* Fecal Fermentation of the APEP-A-b

*In vitro* fermentation of APEP-A-b was carried out according to a previous report (35). Briefly, the basic nutrient growth medium was prepared by adding 2.0 g peptone, 2.0 g yeast extract, 0.1 g NaCl, 0.04 g K_2_HPO_4_, 0.04 g KH_2_PO_4_, 0.01 g MgSO_4_, 0.01 g CaCl_2_, 2.0 g NaHCO_3_, 0.02 g hemin, 0.5 g cysteine-HCl, 0. 5 g bile salt, 0.5 g resazurin, 2.0 mL Tween 80 and 1 mg vitamin K1 into 1 L distilled water and adjusting pH to 7.0 using 0.1 M HCl solution.

Fecal samples were acquired from five SPF mice. Fresh fecal samples were mixed with autoclaved, modified physiological saline solution (cysteine-HCl 0.5 g/L and NaCl 9.0 g/L) to obtain 10% (w/v) fecal suspension. The suspension was centrifuged at 500 rpm for 5 min at 4°C, and the supernatant was used as the fecal slurry for further experiments. The large intestinal digestion was initiated by adding 1.0 mL of the fecal slurry into 9.0 mL of basal nutrient medium containing 100.0 mg the APEP-A-b, and the mixture was incubated at 37°C in an Anaero Pack System (Mitsubishi Gas Chemical Co., Inc., Tokyo, Japan). Under the same conditions, the culture medium without the APEP-A-b served as the control. Digested samples at fermentation times of 0, 6, 12, or 24 h were collected for further study.

### Microbiological Culture-Based Techniques

Standard microbiological culture-based techniques were used to analyze microbiota compositions (36). For this, 0.1 g fresh fecal samples were weighed and diluted in 1 mL of 0.9% NaCl solution and homogenized by using a tissue grinder. The suspension was serially diluted 10-fold with the saline solution down to a 10^-7^ dilution. Then 50 μL of indicated dilutions (**Table 1**) were plated and inoculated on selective agars under aerobic or anaerobic conditions.

**Table 1:** Media and cultivation conditions used for enumeration of microorganisms.

Medium	Cultured microorganism(s)	Dilution ratio	Incubation time
Aerobic cultures			
Eosin-methylene blue agar	Enterobacteriaceae	10^2^, 10^3^	24 h
Bile Esculin Azide Agar	*Enterococcus* spp.	10^3^, 10^4^	48 h
Anaerobic cultures			
LBS agar	*Lactobacillus* spp.	10^5^, 10^6^	48 h
MRS Medium (Li-Mupirocin and Cysteine Hydrochloride Modified MRS Medium Basis)	*Bifidobacterium* spp.	10^4^, 10^5^	48 h
Tryptose Sulfite Cycloserine Agar Base	*Clostridium* *perferingens*	10^1^, 10^2^	24 h

Plates were incubated under anaerobic conditions (AnaeroPack^®^ - Anaero, Mitsubishi Gas Chemical Co.,Inc). All cultured plates were subjected to MALDI-TOF MS measurements, and microorganisms were identified by pattern matching using libraries in the BioTyper 2.0 software program. The number of colony forming units (CFU) were counted and converted to base 10 logarithms for statistical analysis.

### 16S rRNA Gene Pyrosequencing and Bioinformatic Analysis

The CTAB method was used to extract bacterial genome from cecal content of mice. The V3 and V4 regions of bacterial gene 16S rRNA was amplified by PCR using a 341F-806R primer. According to the concentration of PCR products, samples were mixed at the same concentration. PCR products were purified by electrophoresis after thorough mixing, and target bands were recovered. The library was constructed using the NEB Next ^®^ Ultra ™ DNA Library Prep Kit for Illumina (New England Biolabs). After Qubit quantification and library detection, HiSeq was used for on-board sequencing, and raw data was spliced and filtered to obtain Clean Data. Amplicon sequence variants (ASV) clustering and species classification analysis were carried out based on available data, and species annotation was made on the representative sequences of each ASV according to results of ASVs clustering. The QIIME data analysis package was used for 16S rRNA data analysis.

### Cecal Contents SCFAs Quantification

SCFAs were determined as follows: an aliquot of 200 mg of each cecal sample was weighed and suspended in sterile distilled water (1.5 mL). The sample was mixed for 2 h with an orbital shaker, and centrifuged for 10 min at 12000 rpm at room temperature. The upper suspension (1 mL) was transferred to another sterilized centrifugal tube, and the supernatant was combined with 200 μL of 50% H_2_SO_4_ and 2 mL dichloromethane, and then shaken for 10 min and centrifuged at 12,000 rpm for 10 min. The upper suspension was filtered through a 0.22 μm filter before analysis.

SCFA content was measured by using gas chromatography (Shimadzu, Tokyo, Japan) equipped with a DB-FFAP column (30 m×25 mm×0.25 μm). The GC oven temperature was set to 50 °C, increased first to 120 °C at 15 °C/min held for 5 min and then increased to 250°C. The flow rate of nitrogen gas was 30 mL/min, and the injection volume was 1 μL. Contents of total and individual SCFAs were calculated according to calibration curves produced using respective SCFA standards.

### Statistical Analysis

The SPSS statistical software program was used for all data analyses. Results are expressed as the mean ± SD. Differences between experimental groups were analyzed using a one-way analysis of variance (ANOVA) followed by Tukey’s *post hoc* test. Significance was defined as *p*-values < 0.05, <0.01 or <0.001.

## Results

### Preparation of Polysaccharides from *P. eryngii*

Total polysaccharide was extracted from *P. eryngii* fruiting bodies using 0.5 M NaOH, and the yield was 6.0% relative to the dry weight of the material. The homogeneous fraction (APEP-A-b) was obtained first using anion-exchange chromatography, and then purified using gel-permeation chromatography (**Supplementary Figure 1A**). This procedure yielded 36.1% relative to total polysaccharide. APEP-A-b contains 95.2% of total sugar, and the molecular weight of APEP-A-b was ~22.5 kDa as determined by HPGPC (**Supplementary Figure 1B**). Monosaccharide composition analysis showed that APEP-A-b is mainly composed of glucose (90.1%), suggesting it is a glucan. In addition, minor amounts of glucuronic acid, and trace amounts of mannose and galactose were detected.

### Methylation Analysis

To determine linkage types in APEP-A-b, methylation analysis was carried out, with partially methylated alditol acetates (PMAAs) being analyzed by using GC-MS. As shown in **Table 2**, the linkage of glucose in APEP-A-b was mainly in the form of 1,6- and 1,3,6-linkages, indicating that the backbone is 1,6-Glc*p* branched at O-3. Moreover, 1-Glc*p* and 1,3-Glc*p* were detected, most likely as side chains. The degree of branching (DB) in APEP-A-b was ~0.18. From these results, we inferred that APEP-A-b is a branched 1,6-glucan.

**Table 2:** Glycosidic linkages analysis of APEP-A-b by GC–MS.

Methylated sugars	Linkages	Molar ratio (%)	Mass fragments (m/z)
2,3,4-Me_3_-Glc*p*	1,6-	5.0	101,117,129,161,173,189,233
2,4-Me_2_-Glc*p*	1,3,6-	1.1	117,129,159,189,233,261,305
2,4,6-Me_3_-Glc*p*	1,3-	1.0	101,117,129,161,189,233,277
2,3,4,6-Me_4_-Glc*p*	1-	1.1	101,117,129,145,161,205

### NMR Analysis

**Figure 1:**
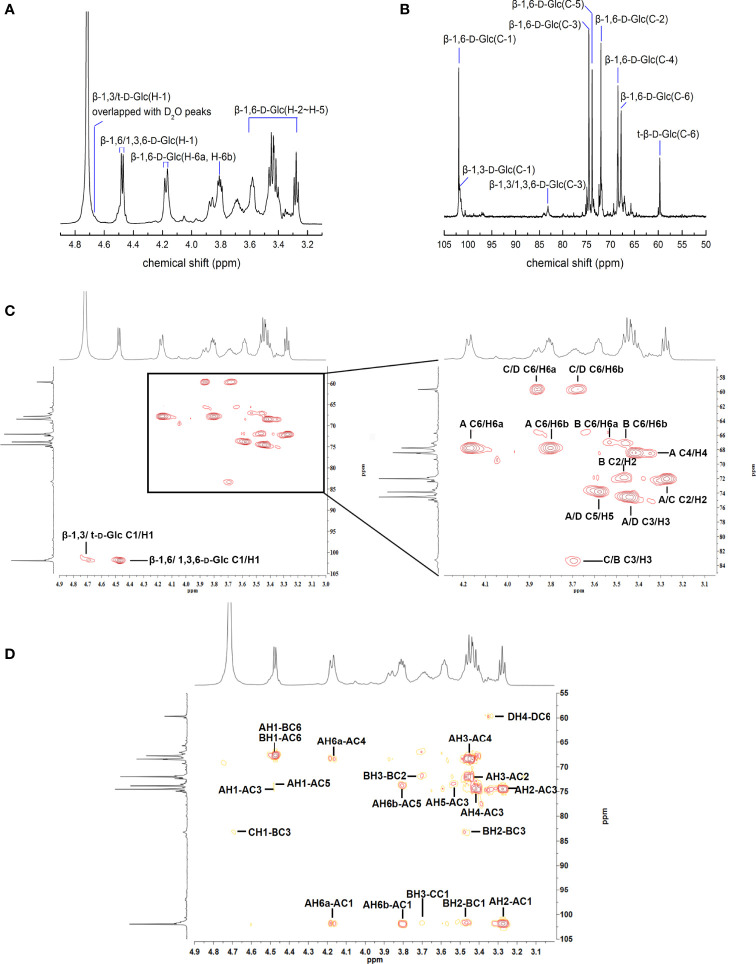
NMR spectra of APEP-A-b. **(A)** 1H NMR spectrum; **(B)** 13C NMR spectrum spectrum; **(C)** HSQC spectrum; **(D)** HMBC spectrum.

The structure of APEP-A-b was analyzed by using ^1^H-^13^C HSQC and HMBC NMR. Resonance assignments are listed in **Table 3**. In the ^1^H-NMR spectrum (**Figure 1A**), anomeric proton signals were found at 4.48 ppm and assigned to anomeric protons of β-D-Glc (37). Chemical shifts in 3.25-4.20 ppm region were assigned to H2-H6 for each sugar residue. In the ^13^C-NMR spectrum (**Figure 1B**), six obvious signals at δ101.92, 72.02, 74.56, 68.42, 73.84 and 67.79 ppm were attributed to C-1, C-2, C-3, C-4, C-5 and C-6 of β-1,6-D-Glc*p* residues, respectively (38). Other proton and carbon signals were assigned based on HSQC spectra (**Figure 1C**). The strong H1/C1 signal at 4.48/101.92 ppm and H6/C6 signal at 4.18;3.80/67.79 ppm were attributed to β-1,6-D-Glc*p*. Peaks at 3.27/72.02 ppm, 3.45/74.56 ppm, 3.43/68.42 ppm, 3.58/73.83 ppm were attributed to H2/C2 ~ H5/C5 of β-1,6-D-Glc*p*, respectively. The downfield shifted resonance at 3.69/83.36 ppm arises from H3/C3 of β-1,3-D-Glc*p* or β-1,3,6-D-Glc*p*, whereas the peak at 3.86;3.68/59.69 ppm is from H6a;b/C6 of the terminal-β-D-Glc*p* residue. Signals corresponding to the sugar sequence in APEP-A-b were present in HMBC spectra. Correlations between C-6 of β-1,6-D-Glc*p* and H-1 of β-1,3,6-D-Glc*p* along with C-6 of β-1,3,6-D-Glc*p* and H-1 of β-1,6-D-Glc*p* indicated that the backbone consists of (1→6)-linked-β-D-Glc residues, whereas peaks for C-3 of β-1,3,6-D-Glc*p* and H-1 of β-1,3-D-Glc*p* along with H-3 of β-1,3,6-D-Glc*p* and C-1 of β-1,3-D-Glc*p* suggested that the β-1,6-D-glucan is branched at O-3 by β-1,3-D-Glc*p*. Other peaks were observed as illustrated in **Figure 1D**. In conjunction with methylation analysis, we propose that the structure of APEP-A-b is a branched β-1,6-D-glucan, substituted at O-3 with β-1,3-D-Glc*p* or a single-unit of β-Glc*p*.

**Table 3:** HSQC spectral assignments of APEP-A-b.

Linkage type	Chemical shift (ppm)					
	H-1C-1	H-2C-2	H-3C-3	H-4C-4	H-5C-5	H-6a; 6bC-6
(A) 6→)-β-D-Glc*p*-(1→	4.48	3.27	3.45	3.43	3.58	4.18; 3.80
	101.92	72.02	74.56	68.42	73.83	67.79
(B) 3,6→)-β-D-Glc*p*-(1→	4.47	3.46	3.69	3.35	3.83	3.86; 3.64
	101.95	71.82	83.36	68.53	74.17	65.59
(C) 3→)-β-D-Glc*p*-(1→	4.69	3.59	3.69	3.41	3.79	3.87; 3.68
	101.78	71.83	83.36	68.43	74.06	59.69
(D) β-D-Glc*p*-(1→	4.69	3.38	3.27	3.58	3.83	3.86; 3.68
	101.78	72.00	74.47	68.49	74.17	59.67

### APEP-A-b Enhances Immunity in Mice

**Figure 2:**
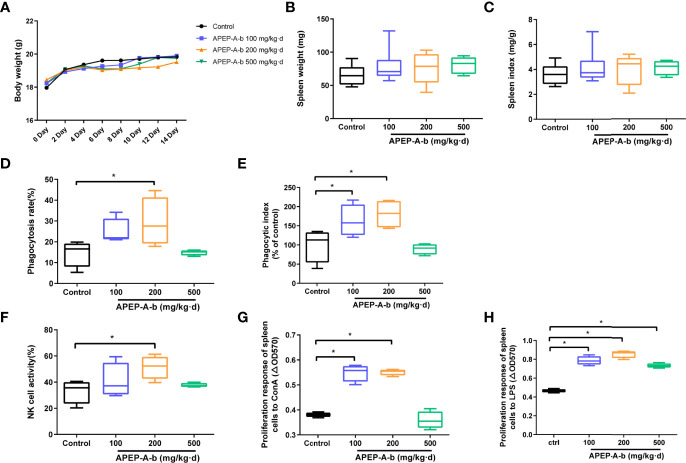
APEP-A-b enhanced immune response in mice. Effects of the APEP-A-b on body weight **(A)**, spleen weight **(B)** and spleen index **(C)**, phagocytosis capacity of peritoneal cavity phagocytes **(D, E)**, NK cell activity **(F)**, ConA-stimulated T cell proliferation **(G)**, LPS-stimulated B cell proliferation **(H)**. Data are represented as the means ± SD (n = 6). *P < 0.05, compared with the Control group.

Immunomodulatory effects of APEP-A-b were assessed in two ways. In the first way, effects on the innate immunity were investigated *via* the killing activity of NK cells, as well as the phagocytic activity of macrophages. The other way was to determine the impact on acquired immunity that was characterized by proliferation of T and B cells. Our results demonstrate that daily oral administration of APEP-A-b does not result in death or significant changes in body weight (**Figure 2A**), spleen weight (**Figure 2B**) or the spleen index (**Figure 2C**), indicating that APEP-A-b is not toxic to mice. Notably, the phagocytosis capacity of peritoneal cavity phagocytes increased by 14.8% compared with that of the Control group in the 200 mg/kg body weight group (**Figure 2D**), whereas 100 or 500 mg/kg body weight groups showed no obvious changes (**Figure 2D**). Consistently, treatment of 100 or 200 mg/kg body weight of APEP-A-b significantly increased the phagocytic index compared with the Control group, whereas 500 mg/kg body weight groups showed no effect on the phagocytic index (**Figure 2E**).

The effect of 200 mg per kg of APEP-A-b on NK cell-mediated cytotoxicity against YAC-1 cells was 51.4%, significantly higher than 33.1% in the Control group (**Figure 2F**). In contrast, in groups treated with the 100 and 500 mg/kg dose of APEP-A-b showed minimal effect on NK cell activity (**Figure 2F**). T and B lymphocytes play important roles in cellular and humoral immunity, respectively. Our results showed that ConA-stimulated proliferation of T splenic lymphocytes increased when APEP-A-b was administered at 100 or 200 mg/kg·d doses (**Figure 2G**). We also observed significant increases in LPS-stimulated proliferation of B splenic lymphocytes when mice were treated with APEP-A-b at doses of 100, 200 and 500 mg/kg (**Figure 2H**). Collectively, our results indicate that APEP-A-b robustly enhances phagocytosis capacity of peritoneal cavity phagocytes, NK cell-mediated cytotoxicity, as well as splenic lymphocyte proliferation in mice. The dose of 200 mg/kg·d exerted the most significant immunomodulatory activity.

### APEP-A-b Enhances Intestinal Immunity in Mice

**Figure 3:**
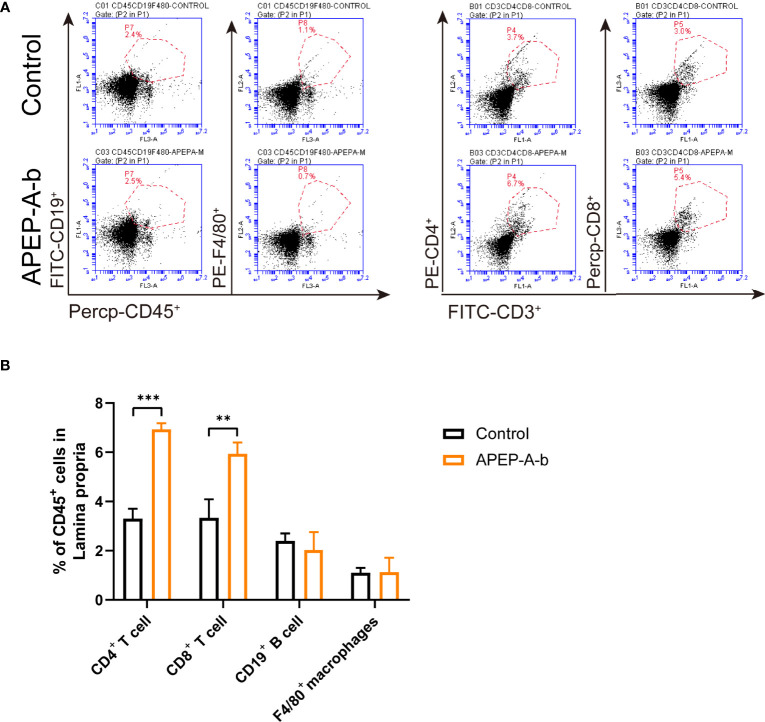
APEP-A-b changed the proportion of immune cells in lamina propria. Flow cytometric analysis of CD4^+^ T cells (CD45.2^+^CD3^+^CD4^+^), CD8^+^ T cells (CD45.2^+^CD3^+^CD8^+^), B cells (CD45.2^+^CD19^+^), macrophages (CD45.2^+^F4/80^+^) and dendritic cells (CD45.2^+^Ly6G^+^) in the lamina propria of Control group (upper panels) and APEP-A-b 200 mg/kg·d group (lower panels). Representative dot plots **(A)** and summary data **(B)** depict of the frequencies of cell subsets. Data are represented as the means ± SD (n = 6). **P < 0.01 and ***P < 0.001 compared with the Control group.

Dietary supplementation has been considered a window of opportunity to modulate intestinal immunity. To further investigate immune responses to APEP-A-b, the proportion of cells in lamina propria were investigated by using flow cytometry. Our results showed that treatment with 200 mg/kg·d APEP-A-b increased the proportion of CD4^+^ and CD8^+^ T cells, but not B cell or macrophages (**Figures 3A, B**).

### Artificial Gastrointestinal Digestions and Fermentation of APEP-A-b

**Figure 4:**
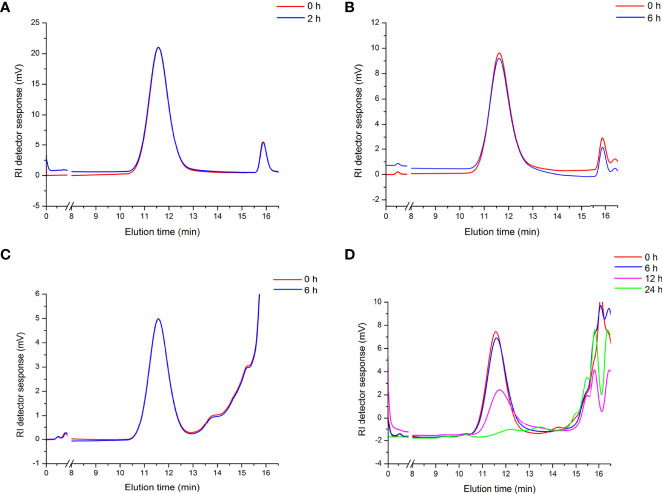
Artificial gastrointestinal digestions and fermentation of APEP-A-b. Molecular weight distributions of the APEP-A-b during saliva **(A)**, gastric **(B)**, small intestinal digestions **(C)** and fermentation **(D)** in different processing times *in vitro*.

To evaluate the mechanism of the immunoregulating effect from APEP-A-b, we used a simulated digestion (simulated saliva, gastric and small intestinal conditions) and fermentation *in vitro*. Our results demonstrate that there are no obvious changes in the molecular weight of APEP-A-b during saliva (**Figure 4A**), gastric (**Figure 4B**) and small intestinal digestions (**Figure 4C**). However, the molecular weight of APEP-A-b did decline upon fecal fermentation (**Figure 4D**), suggesting that AGLP-A-b can be degraded by gut microbiota.

### Effects of APEP-A-b on Bifidobacterium spp., Clostridium Perfringens, Enterobacteriaceae, Enterococcus spp. and Lactobacillus spp.

**Figure 5:**
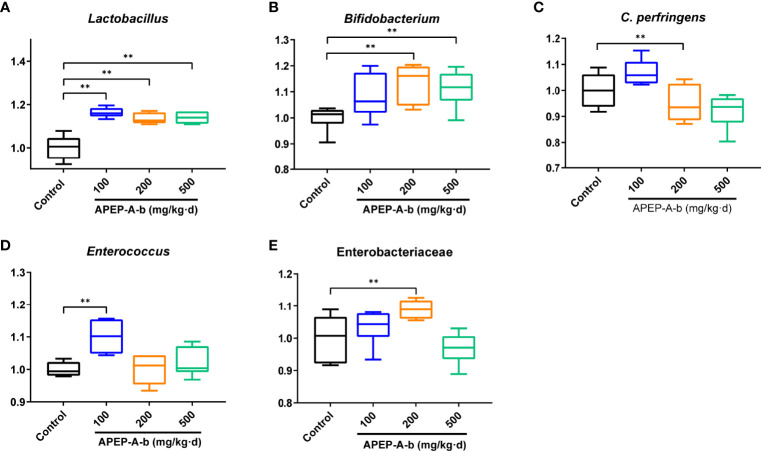
The bacterial counts in faces determined by the culture method. Lactobacillus **(A)**, Bifidobacterium **(B)**, *Clostridium perferingens* **(C)**, Enterobacteriaceae **(D)**, *Enterococcus* **(E)**. Data are represented as the means ± SD (n = 6). **P < 0.01 compared with the Control group.

Based on the above results, we became interested in the effect of APEP-A-b on microbiota. For this, we investigated intestinal microbiota by using a culture- dependent technique. To confirm the types of cultured microorganisms, microbes were subjected to MALDI-TOF MS and identified by pattern matching using libraries in the BioTyper 2.0 software package (**Supplementary Figure 2**). We found that the number of Lactobacillus genus (**Figure 5A**) was significantly increased after treatment with 100, 200 and 500 mg/kg·d APEP-A-b. Similarly, Bifidobacterium genus (**Figure 5B**) was significantly increased in the 200 and 500 mg/kg·d groups, but not in the 100 mg/kg·d group. In contrast, the number of the pathogen *Clostridium perfringens* was robustly decreased in the 200 mg/kg·d group (**Figure 5C**). The number of *Enterococcus* spp. was also robustly decreased in the 200 mg and 500 mg/kg·d APEP-A-b treatment groups (**Figure 5D**), whereas Enterobacteriaceae were robustly increased in the 200 mg/kg·d group (**Figure 5E**). Collectively, our results indicate that APEP-A-b robustly increases the number of probiotics, including *Lactobacillus* genus and *Bifidobacterium* genus, decreased the conditioned pathogen *Clostridium perfringens*. In contrast, APEP-A-b showed differential impact on neutral bacteria, including *Enterococcus* spp. and Enterobacteriaceae.

### Effects of APEP-A-b on Gut Microbiota by 16S rRNA Sequence Analysis

**Figure 6:**
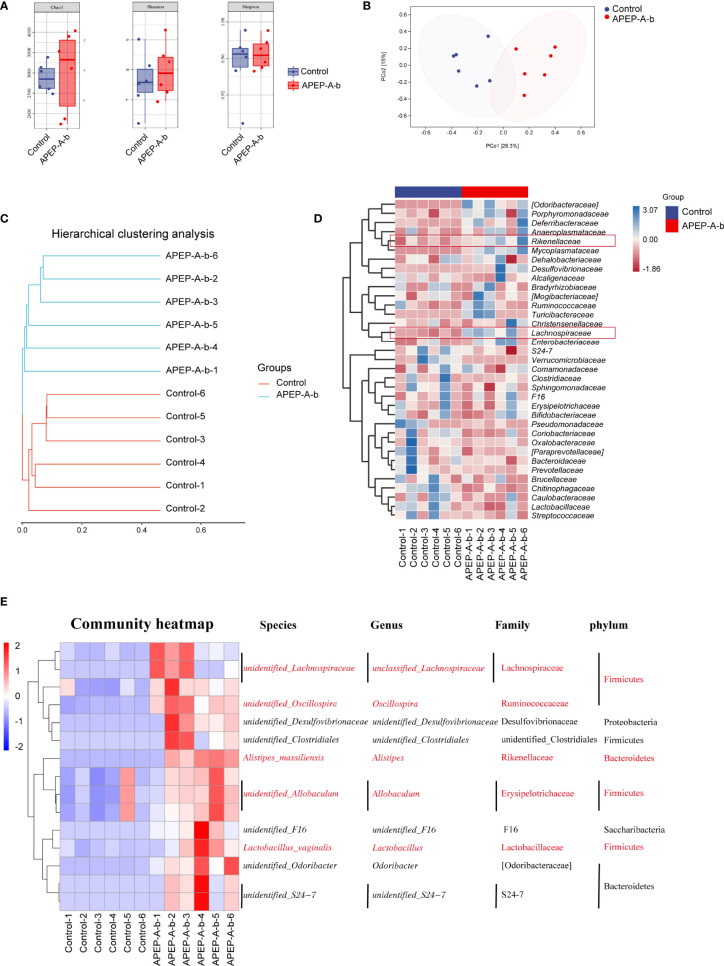
Effects of APEP-A-b on gut microbiota composition tested by 16S rRNA sequencing. 16S rRNA sequencing results were characterized with **(A)** α-diversity, including Chao1, Shannon and Simpson, **(B)** the principal coordination analysis (PCoA) at the amplicon sequence variants (ASV), **(C)** cluster analysis, **(D)** heatmap of microbiota at family level, and **(E)** ASV level, respectively.

16S rRNA sequence analysis showed a dramatic shift in the gut microbiota composition upon 200 mg/kg·d of APEP-A-b treatment. As shown in **Figure 6A**, α-diversity indexes of fecal microbiota (including Chao1 and Shannon) were significantly increased in the APEP-A-b treated group compared to the Control group. A PCA plot was used to characterize relationships among samples based on microorganism composition at the ASV (Amplicon Sequence Variants) level. As shown in **Figure 6B**, samples from the APEP-A-b group were obviously different from the Control group. A similar result was obtained from the hierarchical clustering tree (**Figure 6C**). To obtain the specific composition of microbial communities in the gut, we analyzed differences in the gut microbiota composition at family level. As shown in **Figure 6D**, the abundance of Lachnospiraceae and Rikenellaceae in the APEP-A-b group was increased more than that in the Control group (*P*<0.05). Further analysis was carried out to investigate gut microbiota compositions at the ASV level. The top 15 increased ASVs after orally administration of APEP-A-b is shown in **Figure 6E**. Among them, *unidentified_Lachnospiraceae* (Lachnospiraceae family)*, Oscillospira, Alistipes* (Rikenellaceae family)*, Allobaculum* and *Lactobacillus* are the predominant SCFAs-producing genii, which have been reported to play effective roles in regulating immune responses, maintaining epithelial function, and inhibiting tumor growth (39, 40). Collectively, we conclude that APEP-A-b has potential activity to regulate gut microbiota, and especially to enhance the abundance of SCFAs-producing microbiota.

### APEP-A-b Promotes Production of SCFAs

**Figure 7:**
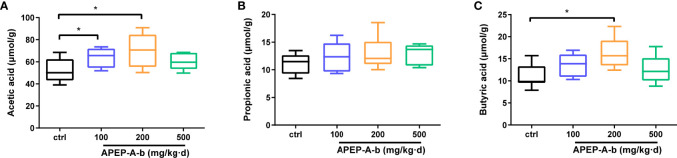
APEP-A-b promotes the production of SCFAs. Effects of the APEP-A-b on the concentration of acetic acid **(A)**, propionic acid **(B)**, and butyric acid **(C)** in cecal contents of mice. Data are represented as the means ± SD (n = 6). *P < 0.05.

Considering changes of SCFAs production upon APEP-A-b treatment, we found that the concentration of acetic (**Figure 7A**) and butyric acid (**Figure 7C**), but not propionic acid (**Figure 7B**), were significantly increased upon treatment with APEP-A-b, especially at the dose of 200 mg/kg of APEP-A-b.

## Discussion

Polysaccharides isolated from *P. eryngii* have attracted considerable attention in recent years due to their low toxicity and broad-based biological activities (8, 41). Previous reports showed that linear (1→6)-β-glucan (8), branched (1→3)-β-glucan (9), 3-O-methylated heterogalactan (6) and heteropolysaccharides (10) have been found in *P. eryngii*. In the present study, we extracted a novel branched (1→6)-β -glucan by using alkaline extraction. We hypothesized that these differences in structures of *P. eryngii* polysaccharides might arise from the source of raw material and the extraction methods. To our knowledge, this is the first report of obtained a branched (1→6)-β-glucan from *P. eryngii*. The use of hot alkali during the extraction process contributed to a strong degradation of glucose chains that resulted in relatively high purity of (1→6)-β-glucan.

The immune system is produced by a network of cells, tissues, and organs that work together to defend the body against attack by “foreign” invaders. Previous work showed that a sulfated polysaccharide (S. PEPS) from *P. eryngii* can inhibit the release of reactive oxygen species (ROS) and nitric oxide (NO) from macrophages (5). KOMAP, a polysaccharide from *P. eryngii*, significantly increased the immune organ index, LPS- or ConA-induced splenocyte proliferation, serum levels of cytokines including IL-2, TNF-α and IFN-γ, as well as the activity of NK and CTL cells in tumor-bearing mice (29). The water-soluble polysaccharide PEPw from the fruiting bodies of *P. eryngii* also showed similar immunomodulating effects in tumor-bearing mice (4).

Macrophages are one of the first lines of defense in the immune system, playing a crucial role in innate and adaptive immunity (42). Natural killer (NK) cells are lymphocytes of the innate immune system that have the ability to kill target cells and produce a series of cytokines (43). The present study showed that APEP-A-b significantly enhanced phagocytosis of peritoneal macrophages, indicating that APEP-A-b can enhance macrophage function in mice. Consistently, NK cell activity in APEP-A-b-treated mice was higher than that in control mice. Lymphocyte proliferation is a response to stimulation induced by antigens or mitogens, which has been widely used to evaluate cellular immune responses. Here, we observed that APEP-A-b (doses of 100 or 200 mg/kg·d) can significantly increase proliferation of splenocytes stimulated with LPS or ConA. Our findings are consistent with previous reports that suggest the commonality of *P. eryngii* polysaccharides in modulating immunity. In previous studies, the dose range from 50 to 800 mg/kg.d of *P. eryngii* polysaccharides was employed for stimulation of the immune system in mice (29, 30). Therefore, here we investigated modulation of immunomodulatory activity using doses of 100, 200 and 500 mg/kg·d of APEP-A-b, and discovered that APEP-A-b promoted immunity in a bell-shaped profile, but not in a concentration- dependent manner. In this regard, the dose of 500 mg/kg·d APEP-A-b did not exert substantial effects, suggesting that there might be some toxicity or other side effects at this highest dose used. This observation may also be explained by considering that APEP-A-b is relatively pure and homogenous, with the effective concentration (200 mg/kg·d) being lower than previously reported (30).

The intestine is the largest digestive and absorbing organ, and is also known as an important mucosal barrier in the human body. T lymphocytes play critical roles in intestinal mucosal immunity. With the regulation of cytokines, naive CD4^+^T cells can differentiate into various Th subsets (44). Cytotoxic CD8^+^ T cells are potent mediators of host protection against disease due to their ability to kill cells that are infected with intra-cellular pathogens (45). Our findings showed that APEP-A-b can significantly increase the proportion of CD4^+^ and CD8^+^ T cells in lamina propria. In this regard, these results suggest that APEP-A-b improves host immunity might by modulating intestinal immunity. Nevertheless, further studies are required to validate this hypothesis.

Gut microbiota is highly correlated with intestinal immunity. Previous reports showed that polysaccharides are capable of regulating host health by influencing the composition of gut microbiota (46). Consistently, gut microbiota and its metabolites can also greatly modulate host responses (47). *P. eryngii* polysaccharides (named PEP) can robustly change fecal microbiota composition, and increase the amount of Porphyromonadaceae, Rikenellaceae, Bacteroidaceae and Lactobacillaceae at the familial level (30). Based on the ability of APEP-A-b to enhance the amount of CD4^+^ T and CD8^+^ T lymphocytes in lamina propria, we investigated whether APEP-A-b could regulate gut microbiota. Therefore, we used culture-based and 16S rRNA sequencing approaches to assess this issue. α-diversity analysis demonstrated that APEP-A-b can enhance microbial community diversity. In addition, β-diversity analysis (including PCA and Hierarchical clustering analysis) revealed that the overall microbial community structure was significantly changed upon treatment with APEP-A-b, with the abundance of Lactobacillus genus, Bifidobacterium genus, Lachnospiraceae and Rikenellaceae in APEP-A-b group being significantly increased. In contrast, the amount of the pathogen *Clostridium perfringens* was significantly decreased. Overall, we concluded that APEP-A-b can increase the abundance of probiotics and decrease pathogen content, suggesting that APEP-A-b is a promising prebiotic. Notably, APEP-A-b affected microbiota composition and its metabolites also in a bell-shaped profile, but not in a concentration-dependent manner. Among them, 200 mg/kg·d of APEP-A-b produced the most substantial effects, consistent with its effect on immunity.

**Figure 8:**
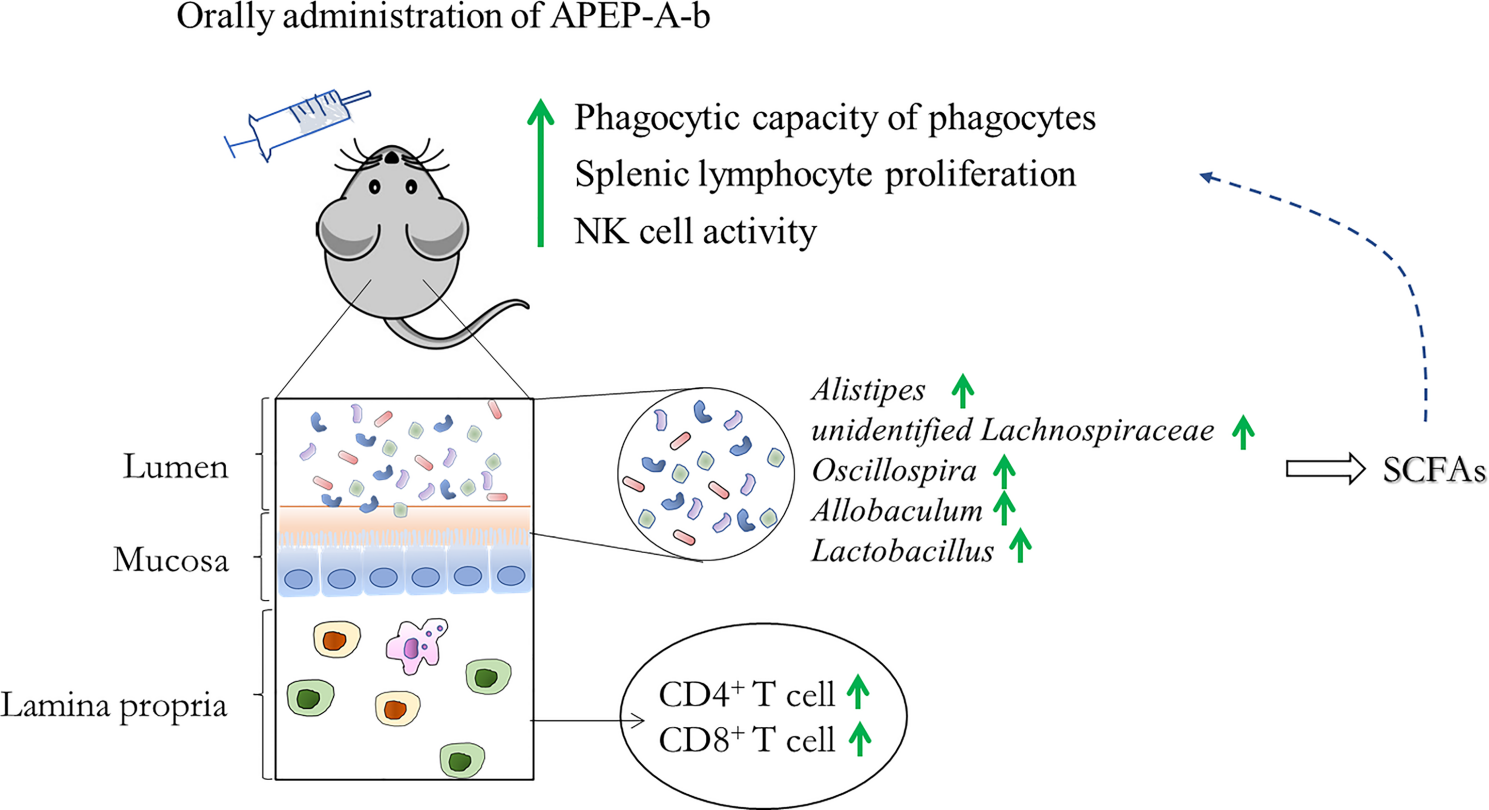
A likely mechanism for the immune regulatory effect from APEP-A-b.

The impact of gut microbiota on the immune system is intricate and partially dependent on the metabolites produced (48). Gut microbiota-derived short chain fatty acids (SCFAs) have been focused upon, due to their profound effect on almost all types of immune cells (49, 50). SCFAs have been shown to have direct effects on differentiation and proliferation of T and B cells (51, 52). *P. eryngii* polysaccharides can be degraded and utilized by the intestinal flora to produce a variety of short-chain fatty acids (SCFAs) (53). Consistent with previous reports, we observed that acetic and butyric acids were significantly upregulated upon administration of APEP-A-b. This strongly suggests that APEP-A-b can increase levels of SCFAs, which might account for its impact on immunity (**Figure 8**). Even though our results are generally consistent with previous reports with β-1,6-glucans, there are some inconsistencies that likely arise from the purity and homogeneity of β-1,6-glucan used in our study. Nevertheless, it is interesting to analyze structure-activity relationships of *P. eryngii* β-1,6-glucans by comparing our results with those in previous studies.

## Conclusion

In the present study, we purified a homogeneous, branched β-1,6-glucan (APEP-A-b) from the fruiting bodies of *P. eryngii* and determined its effects on immunity and gut microbiota in mice. We found that APEP-A-b significantly increases splenic lymphocyte proliferation, NK cell activity, and phagocytic capacity of peritoneal cavity phagocytes. Furthermore, the amount of CD4^+^ and CD8^+^ T cells in lamina propria were significantly increased upon treatment with APEP-A-b. APEP-A-b also increases the amount of SCFAs produced in bacteria to promote production of acetic and butyric acid. Overall, our results suggest that β-1,6-glucan from P. eryngii improves immunity might by modulating the gut microbiota.

## Data Availability Statement

The datasets presented in this study can be found in online repositories. The names of the repository/repositories and accession number(s) can be found below: https://www.ncbi.nlm.nih.gov/, PRJNA812691

## Ethics Statement

The animal study was reviewed and approved by the Ethics Committee of Northeast Normal University.

## Author Contributions

YZ and HC conceived and designed the research. XuW, YQ, YW, XiW, JX and HZ performed the experiments. LS and analyzed the data. YZ, HC, GT, XuW, YQ and DZ wrote and edited the manuscript. All authors reviewed the manuscript and read and approved the final manuscript.

## Funding

This work was supported by grants from the Scientific and Technologic Foundation of Jilin Province (NO.20200708059YY), Jilin Province Development and Reform Commission (NO. 2020C034-6), and the Fundamental Research Funds for the Central Universities (NO.2412020FZ018; NO.2412020QD017).

## Conflict of Interest

The authors declare that the research reported here was conducted in the absence of any commercial or financial relationships that could be construed as a potential conflict of interest.

## Publisher’s Note

All claims expressed in this article are solely those of the authors and do not necessarily represent those of their affiliated organizations, or those of the publisher, the editors and the reviewers. Any product that may be evaluated in this article, or claim that may be made by its manufacturer, is not guaranteed or endorsed by the publisher.
