# Supplementary material for: β-1,6-Glucan From Pleurotus eryngii Modulates the Immunity and Gut Microbiota
Source: Front Immunol. 2022 May 2;13:859923. doi: 10.3389/fimmu.2022.859923 (PMC9108243; doi:10.3389/fimmu.2022.859923)
Supplement: Supplementary file 1 [file DataSheet_1.doc]

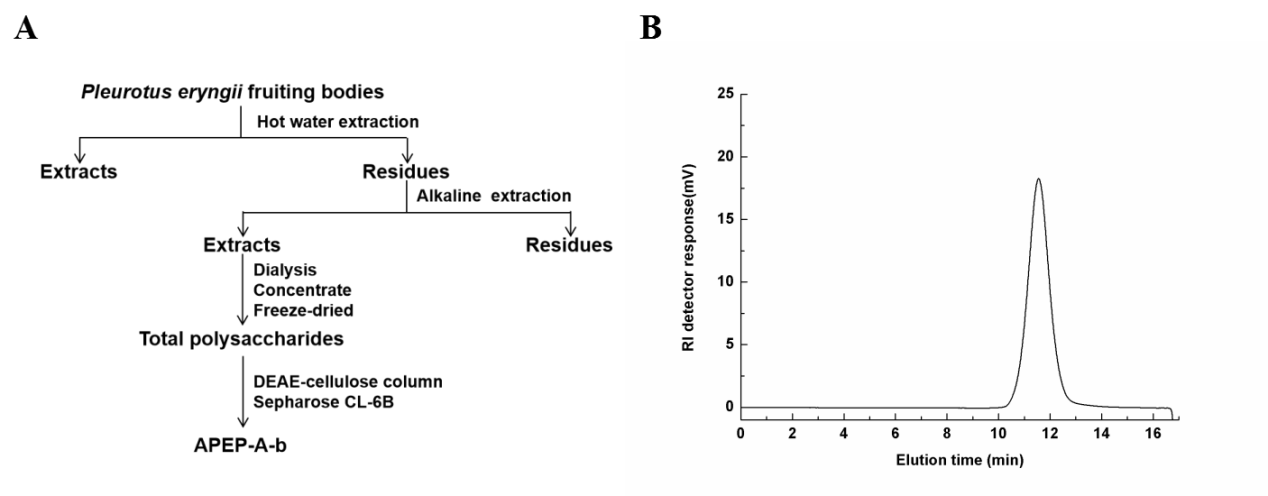


Supplementary Figure 1. Preparation of polysaccharides. (A) Extraction and separation scheme of the polysaccharide. (B) HPGPC elution profiles of the polysaccharide fraction.


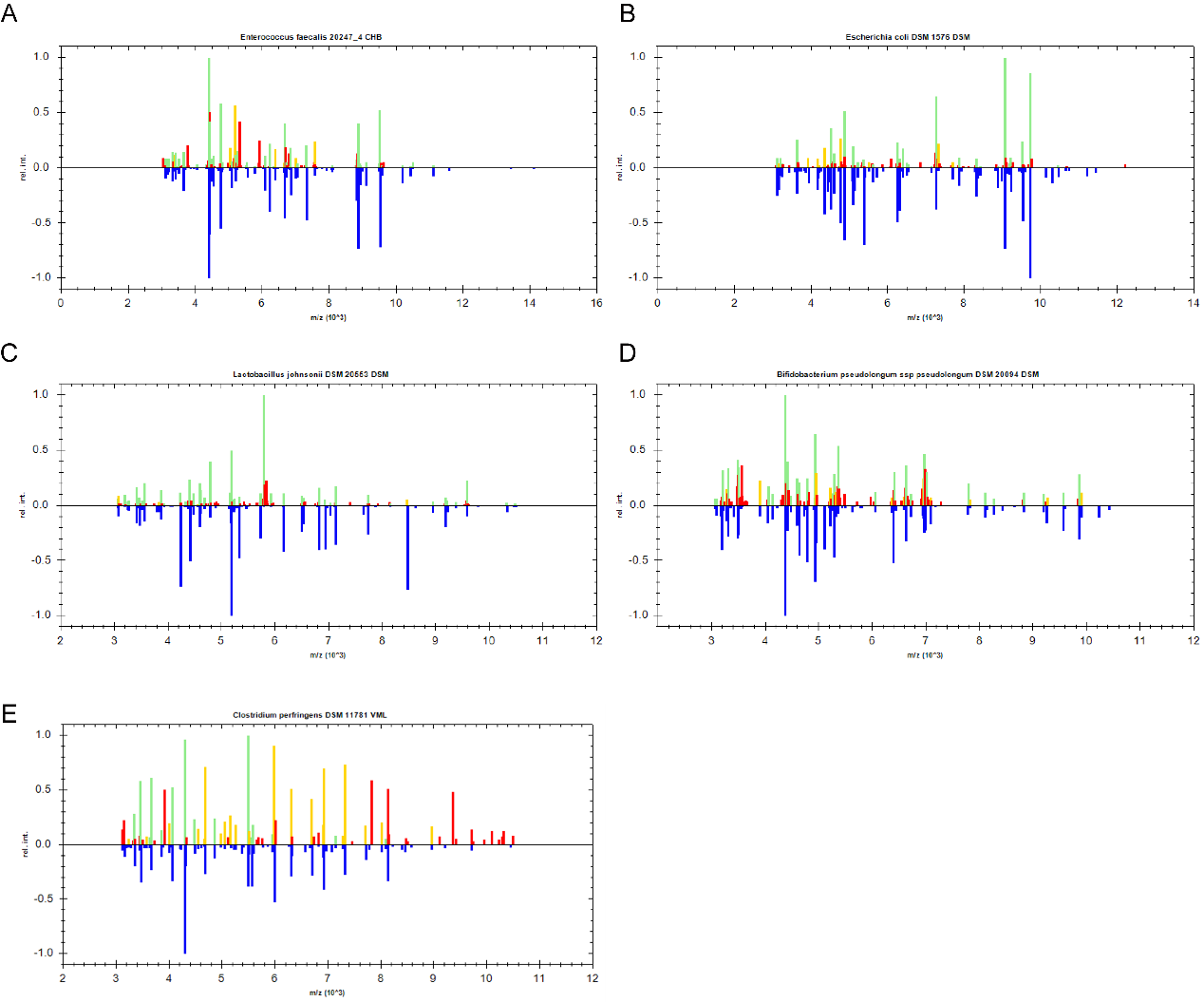


Supplementary Figure 2. The microorganisms were subjected to MALDI-TOF MS measurement and identified by pattern matching by the libraries in the BioTyper 2.0 software. *Enterococcus* (A), Enterobacteriaceae (B), *Lactobacillus* (C), *Bifidobacterium* (D) and *Clostridium perferingens*(E).
